# 6-Hy­droxy-5-[(2-hy­droxy-4,4-dimethyl-6-oxo­cyclo­hex-1-en­yl)(4-nitro­phen­yl)meth­yl]-1,3-di­methyl­pyrimidine-2,4(1*H*,3*H*)-dione

**DOI:** 10.1107/S1600536813028584

**Published:** 2013-10-23

**Authors:** N. Sureshbabu, V. Sughanya

**Affiliations:** a Department of Chemistry, Annamalai University, Annamalai Nagar 608 002, Tamil Nadu, India

## Abstract

In the title compound, C_21_H_23_N_3_O_7_, the pyrimidine­dione ring adopts a screw-boat conformation, whereas the cyclo­hexenone ring adopts an envelope conformation, with the C atom bearing the methyl groups as the flap atom. The dihedral angle between the mean planes of the pyrimidine­dione and cyclo­hexenone rings is 58.78 (2)°. The pyrimidine­dione and cyclo­hexenone rings form dihedral angles of 59.94 (3) and 54.73 (2)°, respectively, with the 4-nitro­phenyl ring. Relatively strong intra­molecular O—H⋯O hydrogen bonds are observed. In the crystal, mol­ecules are linked by C—H⋯O hydrogen bonds, forming a chain along the *c-*axis direction.

## Related literature

For related syntheses, see: Horning & Horning (1946 ▶); Kaupp *et al.* (2003 ▶). For biological and pharmaceutical properties of pyrimidine derivatives, see: Ibrahim & El-Metwally (2010 ▶); Kappe (1993 ▶); Campbell *et al.* (1988 ▶); Elinson *et al.* (2006 ▶); Sun *et al.* (2006 ▶). For bond-length data, see: Allen *et al.* (1987 ▶). For the crystal structure of a related bis­dimedone derivative, see: Sughanya & Sureshbabu (2012 ▶). For the assignment of ring conformations, see: Cremer & Pople (1975 ▶).
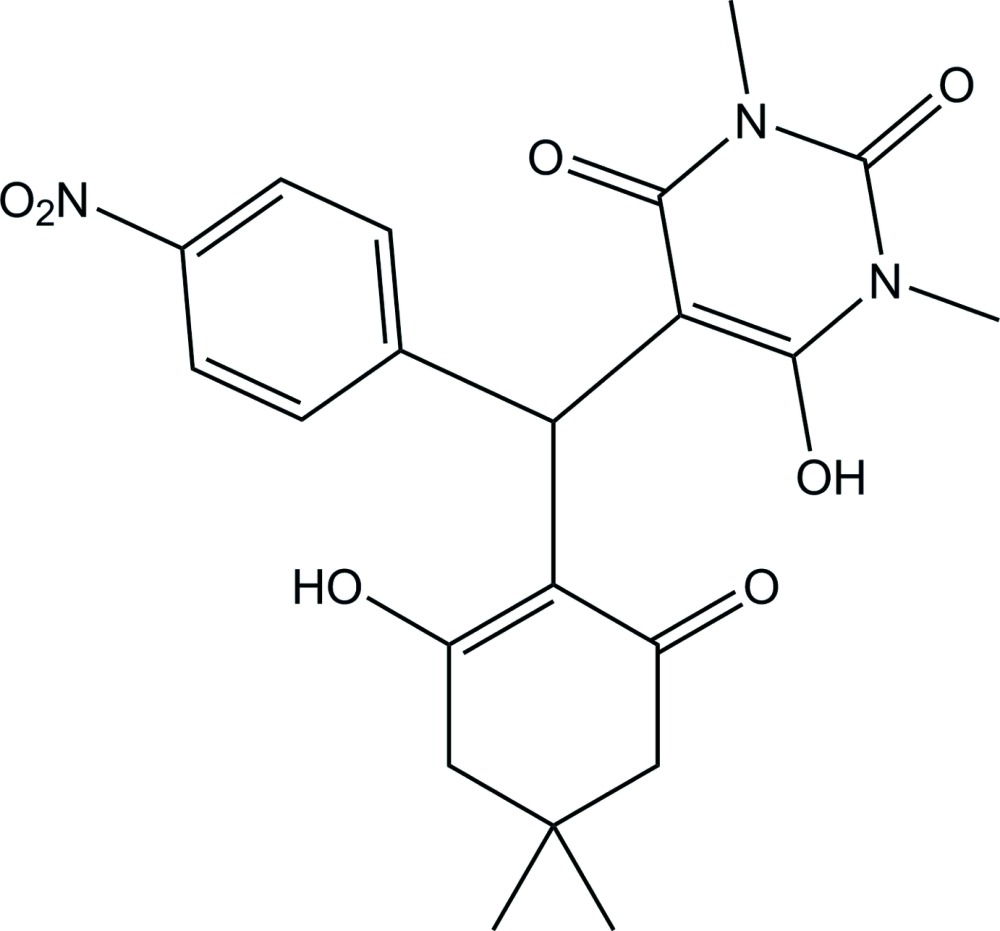


## Experimental

### 

#### Crystal data

C_21_H_23_N_3_O_7_
*M* *_r_* = 429.42
Monoclinic, 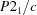

*a* = 12.7470 (2) Å
*b* = 14.0577 (3) Å
*c* = 11.7639 (2) Å
β = 99.752 (1)°
*V* = 2077.55 (7) Å^3^
*Z* = 4
Mo *K*α radiation
μ = 0.10 mm^−1^
*T* = 296 K
0.30 × 0.20 × 0.20 mm

#### Data collection

Bruker Kappa APEXII CCD diffractometer
Absorption correction: multi-scan (*SADABS*; Bruker, 2004 ▶) *T*_min_ = 0.953, *T*_max_ = 0.996
19038 measured reflections
3655 independent reflections
2930 reflections with *I* > 2σ(*I*)
*R*_int_ = 0.026

#### Refinement

*R*[*F* ^2^ > 2σ(*F* ^2^)] = 0.038
*wR*(*F* ^2^) = 0.111
*S* = 1.03
3655 reflections
284 parameters
H-atom parameters constrained
Δρ_max_ = 0.20 e Å^−3^
Δρ_min_ = −0.17 e Å^−3^

### 

Data collection: *APEX2* (Bruker, 2004 ▶); cell refinement: *APEX2* and *SAINT* (Bruker, 2004 ▶); data reduction: *SAINT* and *XPREP* (Bruker, 2004 ▶); program(s) used to solve structure: *SIR92* (Altomare *et al.*, 1993 ▶); program(s) used to refine structure: *SHELXL97* (Sheldrick, 2008 ▶); molecular graphics: *ORTEP-3 for Windows* (Farrugia, 2012 ▶) and *Mercury* (Macrae *et al.*, 2008 ▶); software used to prepare material for publication: *SHELXL97*.

## Supplementary Material

Crystal structure: contains datablock(s) global, I. DOI: 10.1107/S1600536813028584/is5316sup1.cif

Structure factors: contains datablock(s) I. DOI: 10.1107/S1600536813028584/is5316Isup2.hkl

Supplementary material file. DOI: 10.1107/S1600536813028584/is5316Isup3.cml

Additional supplementary materials:  crystallographic information; 3D view; checkCIF report

## Figures and Tables

**Table 1:** Hydrogen-bond geometry (Å, °)

*D*—H⋯*A*	*D*—H	H⋯*A*	*D*⋯*A*	*D*—H⋯*A*
C14—H14*B*⋯O3^i^	0.97	2.57	3.328 (2)	135
C20—H20⋯O7^i^	0.93	2.53	3.153 (2)	125
O2—H2⋯O4	0.82	1.78	2.5932 (18)	172
O5—H5⋯O3	0.82	1.78	2.5863 (18)	167

Symmetry code: (i) 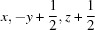.

**Fig. 1.**
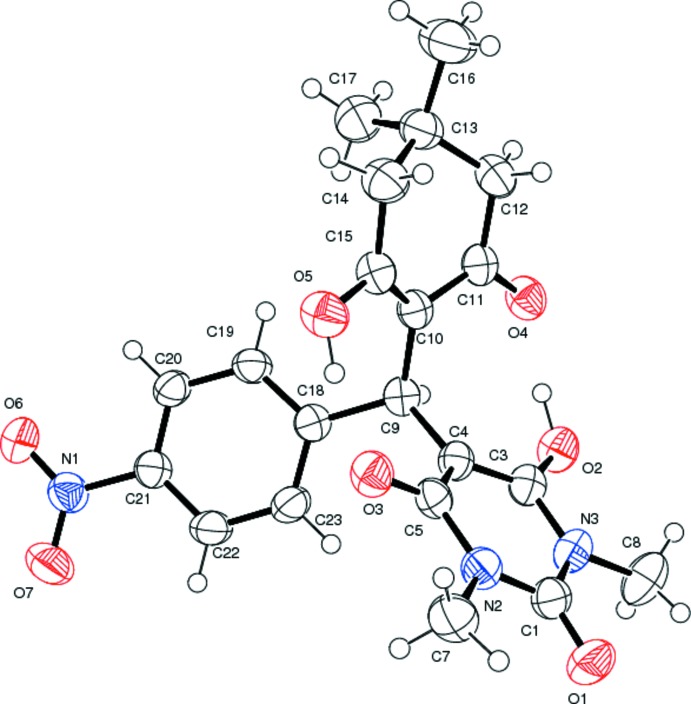
A view of the structure of the title compound. Displacement ellipsoids are drawn at the 30% probability level. Hydrogen atoms are shown as spheres of arbitrary radius.

**Fig. 2.**
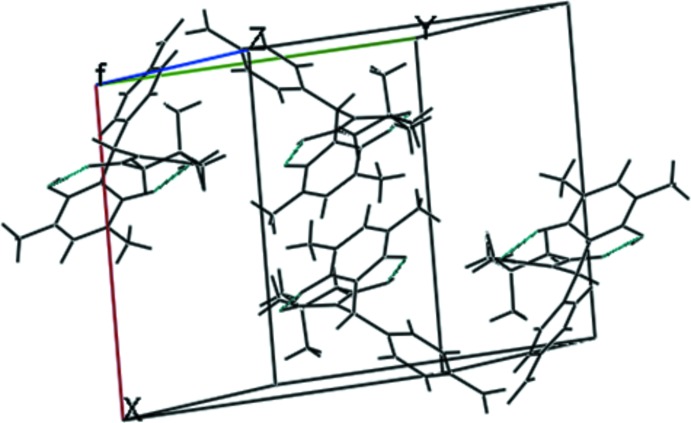
A view of the packing in the crystal structure, showing intramolecular O—H···O hydrogen bonds as dotted lines.

## supplementary crystallographic information

### 1. Comment

Organic compounds containing pyrimidine scaffold as a core unit are important targets and are known to exhibit various biological and pharmaceutical activities (Kappe, 1993; Ibrahim & El-Metwally, 2010). Dihydropyridine derivatives are particularly well known in pharmacology as L-type calcium channel blockers (Campbell *et al.*, 1988; Elinson *et al.*, 2006; Sun *et al.*, 2006).

In the title compound, the bond lengths (Allen *et al.*, 1987) and angles are generally within normal ranges. The pyrimidinedione ring P(N2/C1/N3/C3/C4/C5) and the cyclohexenone ring Q(C10–C15) are not planar with total puckering amplitude Q(T) of 0.0868 (18) Å (for P) and 0.4810 (2) Å (for Q). The pyrimidinedione ring (P) adopts a screw-boat conformation, whereas the cyclohexenone ring (Q) adopts an envelope conformation and atom C13 is described as the flap atom being away from the plane of the ring with deviation 0.334 (2) Å. These conformations can be rationalized by the respective puckering parameters (Cremer & Pople, 1975) φ = 248.7 (12)°, θ = 76.6 (12)° (for P) and φ = -4.90 (3)°, θ = 115.1 (2)° (for Q). The dihedral angle between the mean palnes of the pyrimidinedione ring P and the cyclohexenone plane Q is 58.78 (2)°. The ring P and the plane Q form dihedral angles of 59.94 (3) and 54.73 (2)°, respectively, with the 4-nitrophenyl ring. The hydroxy and carbonyl oxygen atoms face each other and are oriented to allow for the formation of two intramolecular O—H···O hydrogen bonds (Table 1 and Fig. 2) typical for bisdimedone derivatives (Sughanya & Sureshbabu, 2012).

### 2. Experimental

The title compound was prepared in a single stage (Horning & Horning, 1946; Kaupp *et al.*, 2003). A mixture of 4-nitrobenzaldehyde (1.51 g, 10 mmol), 5,5-dimethylcyclohexane-1,3-dione (1.40 g, 8 mmol), 1,3-dimethyl-2,4,6(1*H*,3*H*,5*H*)pyrimidinetrione (1.56 g, 10 mmol) and 20 ml of ethanol was heated to 70 °C for about 10 minutes. The reaction mixture was allowed to cool to room temperature and the resulting title compound was filtered and dried. The yellow crystal used for data collection was obtained by crystallization from ethanol at room temperature,(m.p. 446 K; yield 3.69 g, 86%).

### 3. Refinement

All hydrogen atoms were identified from difference in electron density peaks and subsequently treated as riding atoms with *d*(Csp^2^—H) = 0.93 Å, *d*(C_methyl_—H) = 0.96 Å, *d*(C_methylene_—H) = 0.97 Å, *d*(C_methine_—H) = 0.98 Å, *d*(O—H) = 0.82 Å, and *U*_iso_(H) = x*U*_eq_(C,O), where x = 1.5 for methyl H and 1.2 for all other H atoms.

### Crystal data

**Table:**

C_21_H_23_N_3_O_7_	*F*(000) = 904
*M**_r_* = 429.42	*D*_x_ = 1.373 Mg m^−^^3^
Monoclinic, *P*2_1_/*c*	Melting point: 446 K
Hall symbol: -P 2ybc	Mo *K*α radiation, λ = 0.71073 Å
*a* = 12.7470 (2) Å	Cell parameters from 6971 reflections
*b* = 14.0577 (3) Å	θ = 2.3–31.1°
*c* = 11.7639 (2) Å	µ = 0.10 mm^−^^1^
β = 99.752 (1)°	*T* = 296 K
*V* = 2077.55 (7) Å^3^	Block, yellow
*Z* = 4	0.30 × 0.20 × 0.20 mm

### Data collection

**Table:**

Bruker Kappa APEXII CCD diffractometer	3655 independent reflections
Radiation source: fine-focus sealed tube	2930 reflections with *I* > 2σ(*I*)
Graphite monochromator	*R*_int_ = 0.026
ω and φ scan	θ_max_ = 25.0°, θ_min_ = 2.2°
Absorption correction: multi-scan (*SADABS*; Bruker, 2004)	*h* = −15→15
*T*_min_ = 0.953, *T*_max_ = 0.996	*k* = −16→16
19038 measured reflections	*l* = −13→13

### Refinement

**Table:**

Refinement on *F*^2^	Primary atom site location: structure-invariant direct methods
Least-squares matrix: full	Secondary atom site location: difference Fourier map
*R*[*F*^2^ > 2σ(*F*^2^)] = 0.038	Hydrogen site location: inferred from neighbouring sites
*wR*(*F*^2^) = 0.111	H-atom parameters constrained
*S* = 1.03	*w* = 1/[σ^2^(*F*_o_^2^) + (0.0532*P*)^2^ + 0.7319*P*] where *P* = (*F*_o_^2^ + 2*F*_c_^2^)/3
3655 reflections	(Δ/σ)_max_ < 0.001
284 parameters	Δρ_max_ = 0.20 e Å^−^^3^
0 restraints	Δρ_min_ = −0.17 e Å^−^^3^

### Special details

**Table:**

Geometry. All esds (except the esd in the dihedral angle between two l.s. planes) are estimated using the full covariance matrix. The cell esds are taken into account individually in the estimation of esds in distances, angles and torsion angles; correlations between esds in cell parameters are only used when they are defined by crystal symmetry. An approximate (isotropic) treatment of cell esds is used for estimating esds involving l.s. planes.
Refinement. Refinement of F^2^ against ALL reflections. The weighted R-factor wR and goodness of fit S are based on F^2^, conventional R-factors R are based on F, with F set to zero for negative F^2^. The threshold expression of F^2^ > 2sigma(F^2^) is used only for calculating R-factors(gt) etc. and is not relevant to the choice of reflections for refinement. R-factors based on F^2^ are statistically about twice as large as those based on F, and R- factors based on ALL data will be even larger.

### Fractional atomic coordinates and isotropic or equivalent isotropic displacement parameters (Å^2^)

**Table:**

	*x*	*y*	*z*	*U*_iso_*/*U*_eq_	
C1	0.43994 (13)	0.52617 (14)	0.32945 (15)	0.0463 (4)	
C3	0.31661 (13)	0.58663 (12)	0.44572 (15)	0.0405 (4)	
C4	0.28899 (12)	0.49692 (12)	0.47480 (14)	0.0379 (4)	
C5	0.34740 (13)	0.41934 (12)	0.44258 (14)	0.0393 (4)	
C7	0.47199 (16)	0.35565 (15)	0.32496 (18)	0.0574 (5)	
H7A	0.4211	0.3198	0.2725	0.086*	
H7B	0.5024	0.3158	0.3883	0.086*	
H7C	0.5273	0.3781	0.2855	0.086*	
C8	0.41348 (18)	0.69691 (15)	0.3370 (2)	0.0674 (6)	
H8A	0.4819	0.6971	0.3132	0.101*	
H8B	0.4142	0.7405	0.4000	0.101*	
H8C	0.3600	0.7161	0.2736	0.101*	
C9	0.19863 (12)	0.48072 (12)	0.54145 (14)	0.0367 (4)	
H9	0.1586	0.5406	0.5336	0.044*	
C10	0.23508 (12)	0.46936 (12)	0.67044 (14)	0.0372 (4)	
C11	0.25023 (13)	0.55493 (12)	0.73689 (15)	0.0400 (4)	
C12	0.28119 (16)	0.55040 (13)	0.86545 (16)	0.0503 (5)	
H12A	0.2477	0.6030	0.8990	0.060*	
H12B	0.3576	0.5589	0.8853	0.060*	
C13	0.25105 (16)	0.45824 (14)	0.91944 (16)	0.0518 (5)	
C14	0.29180 (17)	0.37709 (14)	0.85299 (16)	0.0541 (5)	
H14A	0.3685	0.3730	0.8756	0.065*	
H14B	0.2617	0.3180	0.8753	0.065*	
C15	0.26648 (13)	0.38623 (12)	0.72543 (15)	0.0419 (4)	
C16	0.3044 (2)	0.45346 (18)	1.04585 (18)	0.0775 (7)	
H16A	0.3802	0.4564	1.0507	0.116*	
H16B	0.2854	0.3949	1.0791	0.116*	
H16C	0.2809	0.5061	1.0871	0.116*	
C17	0.13041 (19)	0.45314 (18)	0.9135 (2)	0.0700 (6)	
H17A	0.1071	0.5066	0.9533	0.105*	
H17B	0.1123	0.3953	0.9490	0.105*	
H17C	0.0961	0.4543	0.8343	0.105*	
C18	0.11860 (12)	0.40589 (12)	0.48585 (14)	0.0368 (4)	
C19	0.05242 (13)	0.35990 (13)	0.54975 (14)	0.0419 (4)	
H19	0.0575	0.3744	0.6276	0.050*	
C20	−0.02105 (13)	0.29298 (13)	0.50096 (14)	0.0410 (4)	
H20	−0.0648	0.2624	0.5452	0.049*	
C21	−0.02800 (12)	0.27259 (12)	0.38590 (14)	0.0391 (4)	
C22	0.03304 (15)	0.31929 (15)	0.31838 (15)	0.0527 (5)	
H22	0.0258	0.3061	0.2400	0.063*	
C23	0.10532 (15)	0.38612 (15)	0.36873 (15)	0.0514 (5)	
H23	0.1462	0.4188	0.3231	0.062*	
N1	−0.10298 (12)	0.19886 (11)	0.33551 (13)	0.0476 (4)	
N2	0.41894 (11)	0.43692 (11)	0.36855 (13)	0.0441 (4)	
N3	0.38954 (11)	0.60059 (11)	0.37410 (13)	0.0464 (4)	
O1	0.50086 (11)	0.53864 (11)	0.26191 (12)	0.0635 (4)	
O2	0.27934 (10)	0.66554 (8)	0.48297 (11)	0.0500 (3)	
H2	0.2621	0.6561	0.5461	0.075*	
O3	0.33804 (10)	0.33569 (9)	0.47517 (11)	0.0483 (3)	
O4	0.24328 (10)	0.63529 (9)	0.69037 (11)	0.0494 (3)	
O5	0.27781 (11)	0.30563 (9)	0.67125 (11)	0.0527 (3)	
H5	0.2868	0.3171	0.6052	0.079*	
O6	−0.16084 (13)	0.16313 (11)	0.39533 (12)	0.0695 (4)	
O7	−0.10407 (13)	0.17628 (13)	0.23596 (13)	0.0773 (5)	

### Atomic displacement parameters (Å^2^)

**Table:**

	*U*^11^	*U*^22^	*U*^33^	*U*^12^	*U*^13^	*U*^23^
C1	0.0342 (9)	0.0604 (12)	0.0431 (10)	−0.0059 (8)	0.0029 (8)	0.0007 (9)
C3	0.0339 (8)	0.0411 (10)	0.0448 (9)	0.0020 (7)	0.0013 (7)	0.0043 (8)
C4	0.0329 (8)	0.0388 (9)	0.0410 (9)	−0.0012 (7)	0.0038 (7)	0.0008 (7)
C5	0.0344 (8)	0.0411 (10)	0.0408 (9)	−0.0026 (7)	0.0022 (7)	−0.0040 (8)
C7	0.0478 (11)	0.0640 (13)	0.0630 (12)	0.0038 (9)	0.0168 (9)	−0.0149 (10)
C8	0.0674 (14)	0.0561 (13)	0.0821 (15)	−0.0027 (11)	0.0222 (12)	0.0254 (11)
C9	0.0333 (8)	0.0342 (8)	0.0422 (9)	0.0034 (7)	0.0047 (7)	0.0004 (7)
C10	0.0318 (8)	0.0374 (9)	0.0418 (9)	0.0005 (7)	0.0044 (7)	−0.0016 (7)
C11	0.0331 (8)	0.0383 (10)	0.0477 (10)	0.0015 (7)	0.0044 (7)	−0.0010 (8)
C12	0.0535 (11)	0.0468 (11)	0.0480 (10)	−0.0034 (9)	0.0015 (9)	−0.0074 (8)
C13	0.0624 (12)	0.0499 (11)	0.0417 (10)	−0.0017 (9)	0.0050 (9)	−0.0013 (8)
C14	0.0667 (12)	0.0450 (11)	0.0481 (11)	0.0050 (9)	0.0024 (9)	0.0051 (9)
C15	0.0404 (9)	0.0384 (10)	0.0460 (10)	0.0021 (7)	0.0044 (7)	−0.0012 (8)
C16	0.109 (2)	0.0723 (16)	0.0466 (12)	0.0021 (14)	−0.0003 (12)	−0.0014 (11)
C17	0.0708 (14)	0.0786 (16)	0.0646 (14)	−0.0145 (12)	0.0228 (11)	−0.0096 (12)
C18	0.0305 (8)	0.0409 (9)	0.0383 (9)	0.0023 (7)	0.0036 (7)	0.0014 (7)
C19	0.0383 (9)	0.0539 (11)	0.0343 (8)	−0.0025 (8)	0.0085 (7)	−0.0039 (8)
C20	0.0361 (9)	0.0491 (10)	0.0391 (9)	−0.0044 (7)	0.0097 (7)	0.0024 (8)
C21	0.0322 (8)	0.0447 (10)	0.0398 (9)	−0.0003 (7)	0.0045 (7)	−0.0011 (7)
C22	0.0469 (10)	0.0786 (14)	0.0331 (9)	−0.0154 (10)	0.0080 (8)	−0.0053 (9)
C23	0.0448 (10)	0.0719 (13)	0.0382 (10)	−0.0171 (9)	0.0090 (8)	0.0042 (9)
N1	0.0424 (8)	0.0535 (9)	0.0461 (9)	−0.0038 (7)	0.0055 (7)	−0.0050 (7)
N2	0.0367 (8)	0.0493 (9)	0.0472 (8)	−0.0011 (6)	0.0099 (6)	−0.0063 (7)
N3	0.0399 (8)	0.0475 (9)	0.0522 (9)	−0.0033 (7)	0.0092 (7)	0.0095 (7)
O1	0.0520 (8)	0.0836 (11)	0.0597 (8)	−0.0101 (7)	0.0235 (7)	0.0018 (7)
O2	0.0524 (7)	0.0388 (7)	0.0599 (8)	0.0050 (6)	0.0123 (6)	0.0063 (6)
O3	0.0532 (7)	0.0369 (7)	0.0564 (8)	0.0026 (6)	0.0136 (6)	−0.0042 (6)
O4	0.0564 (8)	0.0358 (7)	0.0542 (8)	0.0024 (6)	0.0040 (6)	−0.0025 (6)
O5	0.0673 (9)	0.0386 (7)	0.0513 (7)	0.0102 (6)	0.0072 (6)	−0.0010 (6)
O6	0.0794 (10)	0.0691 (10)	0.0623 (9)	−0.0344 (8)	0.0185 (8)	−0.0005 (7)
O7	0.0736 (10)	0.1064 (13)	0.0537 (9)	−0.0336 (9)	0.0157 (7)	−0.0312 (9)

### Geometric parameters (Å, º)

**Table:**

C1—O1	1.214 (2)	C13—C14	1.523 (3)
C1—N3	1.377 (2)	C13—C16	1.528 (3)
C1—N2	1.378 (2)	C13—C17	1.529 (3)
C3—O2	1.311 (2)	C14—C15	1.486 (2)
C3—C4	1.368 (2)	C14—H14A	0.9700
C3—N3	1.370 (2)	C14—H14B	0.9700
C4—C5	1.408 (2)	C15—O5	1.320 (2)
C4—C9	1.516 (2)	C16—H16A	0.9600
C5—O3	1.249 (2)	C16—H16B	0.9600
C5—N2	1.386 (2)	C16—H16C	0.9600
C7—N2	1.464 (2)	C17—H17A	0.9600
C7—H7A	0.9600	C17—H17B	0.9600
C7—H7B	0.9600	C17—H17C	0.9600
C7—H7C	0.9600	C18—C19	1.382 (2)
C8—N3	1.470 (2)	C18—C23	1.387 (2)
C8—H8A	0.9600	C19—C20	1.382 (2)
C8—H8B	0.9600	C19—H19	0.9300
C8—H8C	0.9600	C20—C21	1.372 (2)
C9—C10	1.518 (2)	C20—H20	0.9300
C9—C18	1.533 (2)	C21—C22	1.370 (2)
C9—H9	0.9800	C21—N1	1.465 (2)
C10—C15	1.363 (2)	C22—C23	1.378 (3)
C10—C11	1.430 (2)	C22—H22	0.9300
C11—O4	1.252 (2)	C23—H23	0.9300
C11—C12	1.498 (2)	N1—O7	1.2112 (19)
C12—C13	1.520 (3)	N1—O6	1.211 (2)
C12—H12A	0.9700	O2—H2	0.8200
C12—H12B	0.9700	O5—H5	0.8200
			
O1—C1—N3	122.09 (18)	C15—C14—H14A	108.6
O1—C1—N2	122.13 (18)	C13—C14—H14A	108.6
N3—C1—N2	115.77 (15)	C15—C14—H14B	108.6
O2—C3—C4	124.97 (16)	C13—C14—H14B	108.6
O2—C3—N3	113.98 (15)	H14A—C14—H14B	107.6
C4—C3—N3	121.04 (16)	O5—C15—C10	123.67 (16)
C3—C4—C5	118.45 (15)	O5—C15—C14	112.99 (15)
C3—C4—C9	121.19 (15)	C10—C15—C14	123.34 (16)
C5—C4—C9	120.36 (15)	C13—C16—H16A	109.5
O3—C5—N2	117.92 (15)	C13—C16—H16B	109.5
O3—C5—C4	124.36 (16)	H16A—C16—H16B	109.5
N2—C5—C4	117.72 (15)	C13—C16—H16C	109.5
N2—C7—H7A	109.5	H16A—C16—H16C	109.5
N2—C7—H7B	109.5	H16B—C16—H16C	109.5
H7A—C7—H7B	109.5	C13—C17—H17A	109.5
N2—C7—H7C	109.5	C13—C17—H17B	109.5
H7A—C7—H7C	109.5	H17A—C17—H17B	109.5
H7B—C7—H7C	109.5	C13—C17—H17C	109.5
N3—C8—H8A	109.5	H17A—C17—H17C	109.5
N3—C8—H8B	109.5	H17B—C17—H17C	109.5
H8A—C8—H8B	109.5	C19—C18—C23	117.61 (15)
N3—C8—H8C	109.5	C19—C18—C9	120.83 (14)
H8A—C8—H8C	109.5	C23—C18—C9	121.44 (15)
H8B—C8—H8C	109.5	C18—C19—C20	121.72 (15)
C4—C9—C10	113.70 (13)	C18—C19—H19	119.1
C4—C9—C18	113.04 (13)	C20—C19—H19	119.1
C10—C9—C18	115.04 (13)	C21—C20—C19	118.56 (15)
C4—C9—H9	104.5	C21—C20—H20	120.7
C10—C9—H9	104.5	C19—C20—H20	120.7
C18—C9—H9	104.5	C22—C21—C20	121.60 (16)
C15—C10—C11	117.41 (15)	C22—C21—N1	120.00 (15)
C15—C10—C9	125.47 (15)	C20—C21—N1	118.40 (15)
C11—C10—C9	116.62 (14)	C21—C22—C23	118.80 (16)
O4—C11—C10	121.82 (16)	C21—C22—H22	120.6
O4—C11—C12	117.81 (15)	C23—C22—H22	120.6
C10—C11—C12	120.28 (15)	C22—C23—C18	121.59 (16)
C11—C12—C13	114.57 (15)	C22—C23—H23	119.2
C11—C12—H12A	108.6	C18—C23—H23	119.2
C13—C12—H12A	108.6	O7—N1—O6	123.01 (16)
C11—C12—H12B	108.6	O7—N1—C21	118.45 (15)
C13—C12—H12B	108.6	O6—N1—C21	118.55 (15)
H12A—C12—H12B	107.6	C1—N2—C5	123.91 (15)
C12—C13—C14	106.95 (16)	C1—N2—C7	117.79 (15)
C12—C13—C16	109.98 (17)	C5—N2—C7	118.24 (15)
C14—C13—C16	109.41 (17)	C3—N3—C1	122.31 (15)
C12—C13—C17	110.12 (17)	C3—N3—C8	120.72 (16)
C14—C13—C17	111.65 (17)	C1—N3—C8	116.91 (16)
C16—C13—C17	108.70 (18)	C3—O2—H2	109.5
C15—C14—C13	114.76 (15)	C15—O5—H5	109.5
			
O2—C3—C4—C5	−170.34 (15)	C4—C9—C18—C19	159.10 (15)
N3—C3—C4—C5	8.5 (2)	C10—C9—C18—C19	26.2 (2)
O2—C3—C4—C9	8.9 (3)	C4—C9—C18—C23	−24.9 (2)
N3—C3—C4—C9	−172.22 (15)	C10—C9—C18—C23	−157.83 (16)
C3—C4—C5—O3	171.47 (16)	C23—C18—C19—C20	3.2 (3)
C9—C4—C5—O3	−7.8 (2)	C9—C18—C19—C20	179.29 (15)
C3—C4—C5—N2	−9.4 (2)	C18—C19—C20—C21	−0.3 (3)
C9—C4—C5—N2	171.30 (14)	C19—C20—C21—C22	−2.4 (3)
C3—C4—C9—C10	−96.64 (18)	C19—C20—C21—N1	177.59 (15)
C5—C4—C9—C10	82.59 (19)	C20—C21—C22—C23	2.1 (3)
C3—C4—C9—C18	129.82 (16)	N1—C21—C22—C23	−177.94 (17)
C5—C4—C9—C18	−51.0 (2)	C21—C22—C23—C18	1.0 (3)
C4—C9—C10—C15	−86.4 (2)	C19—C18—C23—C22	−3.5 (3)
C18—C9—C10—C15	46.2 (2)	C9—C18—C23—C22	−179.63 (18)
C4—C9—C10—C11	85.25 (17)	C22—C21—N1—O7	4.9 (3)
C18—C9—C10—C11	−142.16 (15)	C20—C21—N1—O7	−175.09 (18)
C15—C10—C11—O4	166.07 (16)	C22—C21—N1—O6	−175.25 (18)
C9—C10—C11—O4	−6.3 (2)	C20—C21—N1—O6	4.7 (2)
C15—C10—C11—C12	−10.5 (2)	O1—C1—N2—C5	−177.80 (16)
C9—C10—C11—C12	177.14 (14)	N3—C1—N2—C5	3.8 (2)
O4—C11—C12—C13	159.47 (16)	O1—C1—N2—C7	−0.6 (3)
C10—C11—C12—C13	−23.8 (2)	N3—C1—N2—C7	−179.05 (15)
C11—C12—C13—C14	50.5 (2)	O3—C5—N2—C1	−177.50 (15)
C11—C12—C13—C16	169.25 (18)	C4—C5—N2—C1	3.4 (2)
C11—C12—C13—C17	−71.0 (2)	O3—C5—N2—C7	5.4 (2)
C12—C13—C14—C15	−47.2 (2)	C4—C5—N2—C7	−173.79 (15)
C16—C13—C14—C15	−166.26 (18)	O2—C3—N3—C1	177.86 (15)
C17—C13—C14—C15	73.4 (2)	C4—C3—N3—C1	−1.1 (2)
C11—C10—C15—O5	−165.52 (16)	O2—C3—N3—C8	−5.1 (2)
C9—C10—C15—O5	6.1 (3)	C4—C3—N3—C8	175.86 (17)
C11—C10—C15—C14	14.3 (3)	O1—C1—N3—C3	176.59 (16)
C9—C10—C15—C14	−174.07 (16)	N2—C1—N3—C3	−5.0 (2)
C13—C14—C15—O5	−163.61 (17)	O1—C1—N3—C8	−0.5 (3)
C13—C14—C15—C10	16.5 (3)	N2—C1—N3—C8	177.89 (16)

### Hydrogen-bond geometry (Å, º)

**Table:**

*D*—H···*A*	*D*—H	H···*A*	*D*···*A*	*D*—H···*A*
C14—H14*B*···O3^i^	0.97	2.57	3.328 (2)	135
C20—H20···O7^i^	0.93	2.53	3.153 (2)	125
O2—H2···O4	0.82	1.78	2.5932 (18)	172
O5—H5···O3	0.82	1.78	2.5863 (18)	167

Symmetry code: (i) *x*, −*y*+1/2, *z*+1/2.
